# Yeasts preservation: alternatives for lyophilisation

**DOI:** 10.1007/s11274-012-1118-y

**Published:** 2012-07-07

**Authors:** Loveness K. Nyanga, Martinus J. R. Nout, Eddy J. Smid, Teun Boekhout, Marcel H. Zwietering

**Affiliations:** 1 Institute of Food, Nutrition and Family Sciences, University of Zimbabwe, Harare, Zimbabwe; 2 Laboratory of Food Microbiology, Wageningen University, Wageningen, The Netherlands; 3 CBS-KNAW Fungal Biodiversity Centre, Utrecht, The Netherlands

**Keywords:** Yeasts preservation, Starter culture, Lyophilisation, Rice cake, Plant fibre

## Abstract

The aim of the study was to compare the effect of two low-cost, low technology traditional methods for drying starter cultures with standard lyophilisation. Lyophilised yeast cultures and yeast cultures preserved in dry rice cakes and dry plant fibre strands were examined for viable cell counts during 6 months storage at 4 and 25 °C. None of the yeast cultures showed a significant loss in viable cell count during 6 months of storage at 4 °C upon lyophilisation and preservation in dry rice cakes. During storage at 25 °C in the dark, yeast cultures preserved in dry rice cakes, and lyophilised cultures of *Saccharomyces cerevisiae* and *Issatchenkia orientalis* showed no significant loss of viable cells up to 4 months of storage. Yeast cultures preserved in dry plant fibre strands had the greatest loss of viable count during the 6 months of storage at 25 °C. Preservation of yeasts cultures in dry rice cakes provided better survival during storage at 4 °C than lyophilisation. The current study demonstrated that traditional methods can be useful and effective for starter culture preservation in small-scale, low-tech applications.

## Introduction

The basic concept of microbial preservation of starter cultures is to avoid any change in the genetic, physiological and morphological characteristics of the microorganisms during storage and to promote a complete depression of all metabolic activity (Cheong et al. 2008). Lyophilisation is one of the most successful methods for preserving bacteria, yeasts and sporulating fungi (Spadaro et al. 2010; Tan et al. 2007). This method offers convenience of storage and postage, and it keeps the microorganisms viable for long periods of time (Miyamoto-Shinohara et al. 2006). However, lyophilisation is relatively expensive as it requires sophisticated equipment and adequate power supply.

Most small and medium-sized enterprises (SME’s) in developing countries cannot afford lyophilisation of starter cultures. Traditional methods for starter culture preservation could be an economical and dependable alternative to lyophilisation in low-tech infrastructure conditions. Fermentation of *pito,* a traditional alcoholic beverage brewed by people from the west African sub-region (Demuyakor and Ohta 1993), uses yeast cells as inoculum originating from a previous brew trapped in the interstices of a traditional woven belt (Sefa-Dedeh et al. 1999). In East Asian countries rice starter cakes which contain complex mixtures of fungi are used for rice wine production (Nout and Aidoo 2002). However, the use of traditional methodologies can result in unpredictable fermentation products as the inocula contain uncontrolled mixed microbiota. As a result, even when the fermentation process is successful, its outcome could show considerable variation in product quality. These traditional starter preservation methods could be harnessed for dependable and low-cost preservation of defined fermentation starter cultures. Since the traditional methods are usually economically feasible and can be applied under rural conditions, the basic work flow of these processes is preferably kept intact.

This study was designed to assess two traditional methods for drying defined starter cultures, i.e., stabilization of yeast cultures in plant fibre strands and in rice cakes, for subsequent use in *masau* wine production and compare these methods with lyophilisation. For this case study, we used yeast isolates previously isolated from the traditionally fermented *Ziziphus mauritiana* (*masau*) fruit pulp (Nyanga et al. 2007). In Zimbabwe, the fermented *masau* fruit pulp is subsequently distilled into a spirit called *kachasu* (Nyanga et al. 2008).

## Materials and methods

### Preparation of the inoculum

Cultures used in this study were *Saccharomyces cerevisiae* (strains 38 and 153), *Saccharomycopsis fibuligera* (66) and *Issatchenkia orientalis* (129). These strains were previously isolated from traditionally fermented *masau* fruit pulp (Nyanga et al. 2007) and were maintained routinely at −80 °C in 300 mL L^−1^ glycerol prepared in peptone physiological saline (PPS) [NaCl 8.5 g L^−1^ (Merck, Darmstadt, Germany), neutral peptone 1 g L^−1^ (Oxoid, Basingstoke, UK)].

Yeast cells were grown on Malt Extract Agar (MEA) (Oxoid, Basingstoke, UK) slants at 30 °C for 48 h. A suspension of yeast cells was made by adding 2 mL of sterile PPS onto each pure culture slant. The biomass was gently scraped off the agar by means of an inoculating loop. The yeast cell suspension was then transferred to a sterile tube and used as described below for each preservation method. A fresh yeast culture was made for each method.

### Preservation methods

For each preservation method two independent experiments were performed as described below.

#### Lyophilisation

Yeast suspensions of 1 mL volume were transferred to sterile Eppendorf tubes and centrifuged for 10 min at 2,600×*g* **(**Cheong et al. 2008 **)**. The cells were then washed and centrifuged thrice in 1 mL sterile PPS. The pellet was then suspended in 1 mL sterile solution of 120 g L^−1^ fat free instant milk powder (Clover SA Pty Ltd, Roodepoort, South Africa) prepared in sterile distilled water supplemented with 70 g L^−1^ trehalose (Sigma-Aldrich Co., St Louis, MO, USA) (Cheong et al. 2008; Wiemken 1990; Tan et al. 2007). The yeast-milk suspension (0.2 mL) was transferred to sterile cryotubes. The cryotubes were kept at −58 °C for 3 h and were then lyophilised for 3 h in a lyophilisation apparatus (Edwards Freeze dryer-Modulyo model 4 k, Crawley, West Sussex, England). After freeze-drying the cryotubes were sealed.

#### Rice cakes

Rice flour was made from white polished rice using a pulveriser mill (Siebtechnik GmbH, Mülheim Ruhr, Germany). Portions of rice flour (50 g) were made into a 40 % moisture content dough was made using sterile water according to Dung et al. (2005). The dough from each flask was inoculated with 8 mL of yeast suspension and incubated at 30 °C for 24 h. The inoculated rice dough was then aseptically made into cakes of about 3–4 cm diameter and 5–6 mm thickness. The cakes were aseptically dried at 40 °C in a ventilated oven for 5 h to reach a moisture content of about 4–5 % w/w and then placed in tightly closed containers.

#### Plant fibre strands

The plant fibre belt named *Tafanta* in *Biali* language, and made of twined baobab (*Adansonia digitata*) fibres was obtained from Benin. The plant fibre belt was cut into strands (7–8 cm length and 6 mm thickness) that were wrapped in aluminium foil and sterilized at 121 °C for 15 min. Two hundred mL of Glucose-Yeast extract broth (GYEB) (glucose 100 g L^−1^, yeast extract 0 g L^−1^) was brought into 500 mL volumetric flasks and sterilized at 121 °C for 15 min. Sterile strands were added to GYEB aseptically, inoculated with 1 mL of yeast suspension, whereas a control flask was not inoculated. Broths were fermented for 5 days at 30 °C under non-aerated conditions, plugged with a water-lock. After fermentation the strands were covered and impregnated with sedimented yeast biomass. They were taken out of the flask under aseptic conditions and dried at 40 °C in a ventilated oven for 3 h. The dried and yeast impregnated fibre strands were placed in tightly closed containers.

### Storage conditions

The lyophilised yeast cells and dry rice cakes were stored at 4 °C and room temperature (≈25 °C) in a dark cabinet. The dry plant fibre strands were stored at room temperature (≈25 °C) in a dark cabinet.

### Rehydration and yeast viable cell counts

The survival of the yeast cells was determined by sampling once a week for 1 month and then once every month for 6 months. Viable cells were also determined immediately after each drying method. The number of viable cells was determined as colony forming units per mL (CFU mL^−1^) for the lyophilised yeast cells and as colony forming units per gram (CFU g^−1^) for rice cakes and plant fibre strands.

#### Lyophilisation

The total content of one cryotube was resuspended in 1 mL of PPS and vortexed for 1 min to disintegrate any cell clumps.

#### Rice cakes

A sample of 1 g of rice cake was aseptically transferred into sterile 250 mL bottles containing 99 mL of PPS. The bottles were vigorously shaken at intervals by hand for 10 min to dissolve the cakes.

Plant fibre strands: one strand (0.5 g ± 0.02) was transferred into a sterile 250 mL bottle containing 50 mL of PPS. The bottle was vigorously shaken at intervals by hand for 10 min.

#### Plating

Pour plates were made using glucose peptone yeast extract agar [20 g L^−1^ d-glucose (Merck), 5 g L^−1^ bacto peptone (Oxoid), 5 g L^−1^ yeast extract and 20 g L^−1^ agar (Oxoid); (GPYA)] with appropriate dilutions in duplicate. The inoculated plates were incubated for 48 h at 30 °C and colonies were counted by manual enumeration.

### Survival rate and calculation of D values

Loss of viability was calculated as log N_o_/N_t_, where N_o_ represents the counts of viable microorganisms immediately after drying, and N_t_ the counts of viable microorganisms after a given storage period. Linear regression analysis was carried out on numbers of surviving cells at each storage temperature versus sampling time point. The negative reciprocal of the slope of regression lines was calculated to obtain D values (months). D is the time of the first decimal reduction (i.e., time required to reduce the population by 1 log unit from initial level at *t* = 0).

### Statistical analysis

The analytical data were analysed using the statistical program SPSS16.0 for Windows (Apache Software Foundation, USA) and the one-way ANOVA model was used applying the LSD test to evaluate significant differences among means.

## Results and discussion

**Fig. 1:**
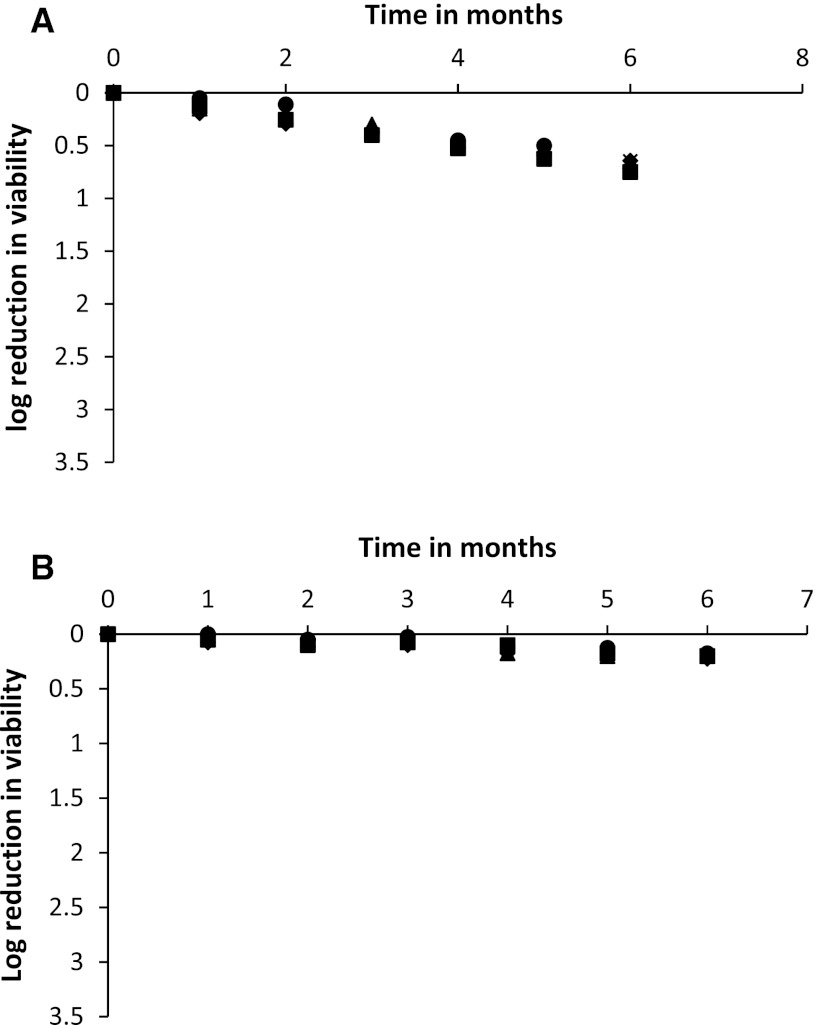
Log reduction in viable counts of each yeast species in lyophilised (**a)** and dry rice cake (**b**) cultures during 6 months storage at 4 °C, *S. cerevisiae* (38), *S. cerevisiae* (153), *I. orientalis* (129) and, *S. fibuligera* (66)

Starter culture preservation, maintenance and distribution are important as the quality of the final fermented product strongly depends on the preservation technologies employed that are required to guarantee long-term maintenance of stable cultures in terms of viability and activity. This demands logistic infrastructure and economic affordability particularly for small-scale, low-tech applications. Thus, in this study we compared the survival of yeast starter cultures preserved by two low-tech traditional methods (i.e., stabilization of yeast cultures in plant fibre strands and in rice cakes), with lyophilisation. Lyophilised yeast cultures and yeast cultures preserved in dry rice cake and dry plant fibre strand were examined for viable cell counts during 6 months storage at 4 and 25 °C in a dark cabinet. During storage at 4 °C, cultures of the different yeast species all showed a similar survival behaviour with a viable cell count reduction of <1 log unit after 6 months storage upon either lyophilisation or drying in rice cakes (Fig. 1). Interestingly, dry rice starter cakes showed minimal losses of viable counts for all strains (viz. between 0.2 and 0.3 logs) as compared to lyophilised cultures, which suffered higher losses of viable counts (viz. between 0.6 and 0.8 logs). This observation was supported by the calculated D values ranging from 28 to 40 months (Table 1), which clearly showed that yeast cells preserved in dry rice cakes survived significantly (*p* < 0.05) better than lyophilised yeast cells which had D values ranging from 8 to 10 months. Cultures of the yeast species showed different D values. For the yeast cultures preserved in dry rice cakes, *S*. *fibuligera* had the highest D value and *S*. *cerevisiae* strain 153 had the lowest value. Lyophilised yeast cultures of *S*. *cerevisiae* strain 38 and *I*. *orientalis* shared the highest D value followed by *S*. *fibuligera* and lastly *S*. *cerevisiae* strain 153.

**Table 1:** Estimated D values (months) of yeast strains preserved by lyophilisation, in dry rice cakes and dry fibre strands stored at 4 and 25 °C

Preservation method and yeast strains		Storage temperature Linear regression D value^1^	
	Strain no.	4 °C	25 °C
Lyophilised			
*S. cerevisiae*	38	10 ± 0.7^a2^	4 ± 0.6^a^
*S. cerevisiae*	153	8 ± 0.96^b^	4 ± 0.11^a^
*I. orientalis*	129	10 ± 0.47^a^	3 ± 0.31^b^
*S. fibuligera*	66	9 ± 1.02^c^	3 ± 0.01^b^
Dry rice cakes			
*S. cerevisiae*	38	33 ± 1.91^d^	5 ± 1.43^d^
*S. cerevisiae*	153	28 ± 1.42^e^	5 ± 1.68^d^
*I. orientalis*	129	31 ± 1.66^f^	4 ± 0.98^e^
*S. fibuligera*	66	40 ± 1.96^i^	5 ± 0.87^d^
Dry plant fibre strands			
*S. cerevisiae*	38	ND	2 ± 0.0^f^
*S. cerevisiae*	153	ND	2 ± 0.09^f^
*I. orientalis*	129	ND	2 ± 0.15^f^
*S. fibuligera*	66	ND	2 ± 0.0^f^

^1^D values ± standard deviation shown are means of two replicate experiments, the enumeration was done in duplicate

^2^Means in the same column with same letter are not significantly different according to the LSD at 0.05 level

*ND* not determined

**Fig. 2:**
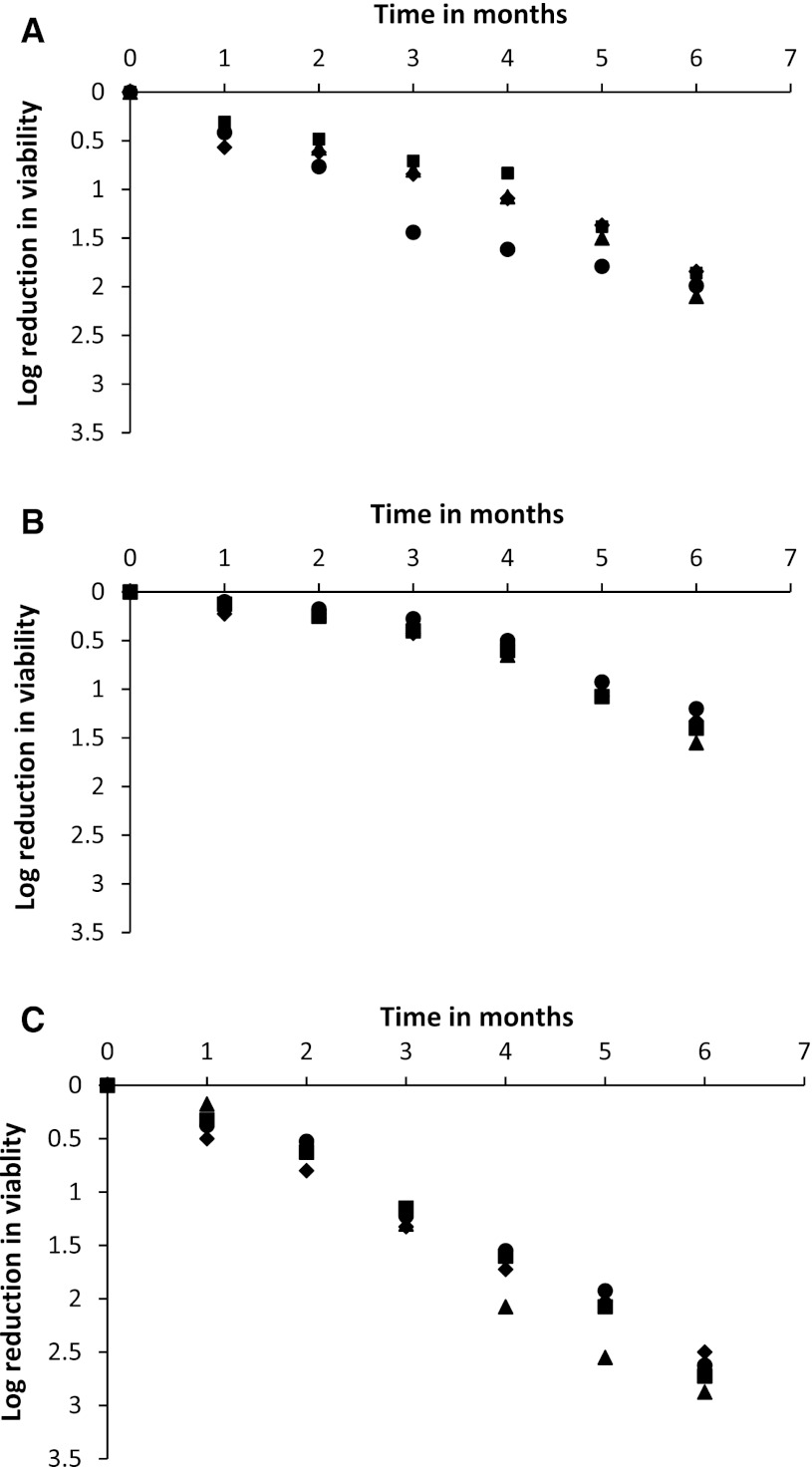
Log reduction in viable count of each yeast species in lyophilised (**a**), dry rice cake (**b**) and dry plant fibre strand (**c**) cultures during 6 months storage at 25 °C, *S. cerevisiae* (38), *S. cerevisiae* (153), *I. orientalis* (129) and, *S. fibuligera* (66)

There was a significant (*p* *<* 0.05) decrease in viable counts of yeast cultures during the six months of storage for all preservation methods for yeasts stored at 25 °C (Fig. 2). It was noted that yeast cultures preserved in dry rice cakes had the best retention of viable cell counts with no significant loss up to 4 months of storage for all the strains. Lyophilisation was second best with *S. cerevisiae* (strains 38 and 153) and *I. orientalis* cultures showing no significant decrease in viable cell counts up to 4 months. On the other hand, lyophilised *S*. *fibuligera* cultures performed differently showing a slight loss in viable cell counts during 3 months of storage. Yeast cultures preserved in dry fibre strands suffered the greatest loss of viable counts as there was significant decrease in viable cell count (between 1.2 and 1.3 log CFU g^−1^) after 3 months of storage. The D values of the yeast cultures preserved in dry plant fibre strands were also lower compared to those obtained from lyophilised cultures and cultures preserved in dry rice cakes.

The results indicated that survival of the yeasts was better at 4 than at 25 °C for both the lyophilised cultures and yeast cultures preserved in dry rice cakes. According to Spadaro et al. (2010) this could be due to the fact that low temperature helps to keep the metabolic activity at a low level which is expected to contribute to an increase in storage and shelf-life. Better survival of lyophilised micro-organisms stored at 4 °C than at 25 °C was reported in several studies (Abadias et al. 2001; De Valdez and Diekmann 1993; Li and Tian 2007; Spadaro et al. 2010). Loss of viability during storage of dried cultures of microbial cells has been attributed to lipid oxidation of cell membrane fatty acids (Coulibaly et al. 2009). This would be initiated by loss of water that increases the ionic concentration, which can lead to the formation of reactive oxygen species. Subsequently, these oxygen species can damage proteins, modify nucleobases and sugars in deoxyribonucleic acid and eventually cause lipid oxidation.

Skimmed milk and trehalose were used in this study as cryoprotectants for the yeast cultures during lyophilisation. The combination of skimmed milk with disaccharides has been reported to improve the viability and stability of micro-organisms during lyophilisation and storage (Li and Tian 2007; Tan et al. 2007). Direct interactions between sugar molecules and membrane phospholipids, or sugar molecules and proteins, and vitrification of sugars in the dry state are thought to be the main protective mechanisms (Crowe et al. 1998).

In this study, dry rice cakes provided the best retention of viable cell counts for the yeast cultures. Rice contains starch, which comprises amylose and amylopectin that could have provided hydroxyl groups as reactive groups for attachment to the yeast cells and possibly by the formation of a glass structure, hence protecting the yeast cells from damage. Mazzobre et al. (1999) reported that saccharides are able to form a glassy structure, in which the sensitive components of the membrane are embedded.

The preservation of starter cultures by a simple and adoptable technology using locally available substrates such as grains and pulses that are familiar to the consumers, would be compatible with the existing low level of technology (Isu and Abu 2000) in most developing countries.

## Conclusion

Dry rice cake cultures retained viable cell counts significantly better as compared to lyophilised cultures. Traditional culture preservation methods, such as those using dry rice cakes and dry plant fibres can be applied to preserve defined starter cultures for traditional fermented foods. In order to safeguard the quality of the preserved yeast, the preservation should be carried out under hygienic and controlled conditions. The use of dry rice cakes and plant fibre strands as preservation methods are of interest in small-scale, low-tech applications.
